# Incidence of Rotavirus-Related Hospitalizations in an Italian Southern Region from 2015 to 2021

**DOI:** 10.3390/diseases12010026

**Published:** 2024-01-17

**Authors:** Giuseppe Di Martino, Fabrizio Cedrone, Michela D’Addezio, Camillo Odio, Pamela Di Giovanni, Edoardo Trebbi, Livia Tognaccini, Ferdinando Romano, Tommaso Staniscia

**Affiliations:** 1 Department of Medicine and Ageing Sciences, “G. d’Annunzio” University of Chieti-Pescara, 66100 Chieti, Italy; pamela.digiovanni@unich.it (P.D.G.); tommaso.staniscia@unich.it (T.S.); 2 Unit of Hygiene, Epidemiology and Public Health, Local Health Authority of Pescara, 65100 Pescara, Italy; michela.daddezio@asl.pe.it; 3 Hospital Management, Local Health Authority of Pescara, 65100 Pescara, Italy; cedronefab@gmail.com; 4 Digital Health Unit, Department of Health, Abruzzo Region, 65100 Pescara, Italy; camillo.odio@regione.abruzzo.it; 5 Department of Public Health and Infectious Diseases, “La Sapienza” University of Rome, 00100 Rome, Italy; edoardo.trebbi@uniroma1.it (E.T.); livia.tognaccini@uniroma1.it (L.T.); ferdinando.romano@uniroma1.it (F.R.)

**Keywords:** rotavirus, hospitalization, epidemiology, public health, Italy

## Abstract

(1) Background: Rotavirus is one of the leading causes of severe diarrhea and dehydration in infants and young children worldwide. The economic and social burden of rotavirus-related hospitalizations, particularly among children, remains a pressing concern for healthcare systems across the globe. Healthcare infrastructure and access to medical care can vary significantly within the region. Differences in the availability of healthcare facilities and the quality of care may influence the management and outcomes of rotavirus cases. (2) Methods: This was a retrospective study performed in the Abruzzo region, Italy. The study considered all hospitalization due to rotavirus gastroenteritis that occurred in the Abruzzo region from the year 2015 to 2021. Data were extracted from the hospital discharge records. The trend in hospital admissions, hospitalization costs and length of stay were evaluated and analyzed. (3) Results: A total of 664 admissions were reported during the study period. The incident rate grew till year 2019 with an annual percentage change of +13.9% (95%CI 12.6–15.2, *p* < 0.001). During the pandemic years, the incident rate showed a significant decrease with an annual percentage change of 12.5% (95%CI 15.5–9.3, *p* = 0.004). The length of stay of admissions was significantly higher among patients aged less than 1 year. (4) Conclusions: Rotavirus admission represents a heavy burden even in a high-income country such as Italy. These findings have the potential to inform targeted public health interventions, including vaccination strategies, and improve the overall well-being of children.

## 1. Introduction

Rotavirus, a highly contagious virus primarily affecting infants and young children, continues to pose a significant public health challenge worldwide [1,2]. Rotaviruses are RNA viruses structured by three concentric capsids that include a genome of eleven segments of double-strained RNA. Ten different rotavirus types (A–J) have been classified on the basis of antigenic and genomic differences [1]. Species A rotaviruses are among the most common causes of infections among children worldwide, and they are also classified into different genotypes: in particular, six strains of species A rotavirus generally account for over 90% of globally circulating species [1]. Generally, rotavirus gastroenteritis (RV-GE) affects children aged less than 5 years. In fact, the majority of children younger than 5 years have been infected by RV at least once [3]. RV-GE can lead to a heterogenic spectrum of disease, varying from mild gastroenteritis to dehydration, electrolyte imbalance and shock [4], accounting fore more than 200,000 deaths worldwide, particularly when the infection occurs during the first year of life [5]. Also, patients infected by the emerging G9 and G12 strains had more severe acute gastroenteritis (AGE) than those with other genotypes [6]. Despite progress in reducing the burden of diarrhea morbidity, thanks to simple interventions such as the use of oral rehydration solution, the use of zinc supplements, breastfeeding and adequate complementary feeds, and improvements in water hygiene and sanitation, the mortality due to RV infection remains a public health threat in low-income countries [6].

Despite that, rotavirus gastroenteritis is not only a burden in developing countries but is also one of the most frequent causes of pediatric admissions in industrialized countries [3]. The economic and social burden of rotavirus-related hospitalizations, particularly among children, remains a pressing concern for healthcare systems across the globe [7]. In Italy, as in many other countries, rotavirus is one of the leading causes of severe gastroenteritis in pediatric populations, resulting in substantial healthcare expenditures and potential long-term health consequences [8,9,10].

The epidemiology of rotavirus infection varies geographically and temporally, making it essential to conduct region-specific studies to understand the local impact fully [11]. This study focuses on a southern region of Italy, characterized by its unique demographic, socio-economic and environmental factors, which may influence rotavirus infection rates and subsequent hospitalizations among children.

Southern Italy, known for its Mediterranean climate, has a distinctive population distribution, with a higher concentration of residents in urban areas compared to the northern regions. In low-income countries, the combination of a densely populated urban environment and limited access to clean water and sanitation services in certain communities may enhance the risk of viral transmission [11]. In high-income countries such as Italy, despite the high socioeconomic status and the high level of sanitation, rotavirus frequently spread among infants, particularly during the winter season, as well as in other Mediterranean countries [2].

In Italy, healthcare infrastructure and access to medical care can vary significantly within the region. Differences in the availability of healthcare facilities and the quality of care may influence the management and outcomes of rotavirus cases. Moreover, vaccination coverage rates can vary, impacting the level of immunity within the pediatric population [12]. At present, Italy has approved two oral live attenuated rotavirus vaccines in order to avoid rotavirus infection: RotaTeq^®^ (MSD Canada Inc., Rahway, NJ, USA) and Rotarix^®^ (GlaxoSmithKline, Brentford, UK). RotaTeq^®^ is a 5-valent human–bovine reassortant vaccine and its administration schedule requires three doses to confer immunization from the third month of life, with a minimum gap of one month between each dose. On the other hand, Rotarix^®^ is a monovalent human rotavirus vaccine and its vaccination schedule is organized into two doses distanced at least one month from each other. Vaccine administration should be started between 6 weeks and 12 weeks of age for RotaTeq^®^, and between 6 weeks and 15 weeks of age for Rotarix^®^. In the Abruzzo region, vaccination was offered from the year 2017 on the direct request of parents. Understanding these local healthcare disparities is crucial for implementing targeted interventions to reduce rotavirus-related hospitalizations. The Abruzzo region in the year 2021 reported a vaccination coverage against rotavirus lower than the national result, with a vaccination rate of 58.72% for newborn babies, against the mean national rate of 70.40% [13]. Real-world evidence has confirmed the findings of randomized clinical trials [14] showing a sustained protection against rotavirus disease, especially against severe form of gastroenteritis.

Unlike several other countries, Italy lacks a specific surveillance system focused on rotavirus infections. Despite the introduction of mass vaccination with heterogenous patterns across Italian regions, epidemiological data on rotavirus infection in the Italian context remain poor. For this reason, hospital discharge records (HDRs) represent a valid and standardized tool to evaluate the impact of several diseases on healthcare services, particularly infectious diseases. These records can help in the evaluation of the impact of severe rotavirus cases, giving information on the circulation of the virus and its impact on healthcare services. This study aims to provide a comprehensive analysis of rotavirus infection-related hospitalizations among children in the Abruzzo region of Southern Italy. By examining factors such as age, gender and clinical outcomes, we seek to elucidate the epidemiological landscape of rotavirus in this unique context. Our findings have the potential to inform targeted public health interventions, including vaccination strategies, and to improve the overall well-being of children in the region. No previous studies have been conducted in this region on rotavirus infection, and this study also reports one of the most updated analyses on this topic in the Italian context. This research aligns with the global effort to reduce the burden of rotavirus-related hospitalizations, moving us closer to achieving improved child health outcomes and reduced healthcare costs.

## 2. Materials and Methods

### 2.1. Study Design and Participants

The present study is a retrospective analysis performed in the Abruzzo region, Italy. Abruzzo is a southern Italian region counting more than 1.2 million inhabitants. Its healthcare service is organized into four different local health authorities (LHAs) [15]. Data regarding all hospital admissions that occurred in the Abruzzo region were evaluated from the HDRs of the Abruzzo region. HDRs consider all hospitalizations that occurred during the study period 2015–2021, both for ordinary admissions and daily hospitalizations. In addition, admissions in other Italian regions involving inhabitants from Abruzzo were included in the analysis. Each HDR included:

- information on patient demographic characteristics, such as age, gender, city of residence and marital status;
- the diagnosis-related group (DRG) code used to classify the admission;
- up to six diagnoses (one principal diagnosis and up to five comorbidities);
- up to six possible procedures performed during the hospital stay.

Diagnoses and procedures were coded following the International Classification of Disease, 9th Revision, Clinical Modification (ICD-9-CM), the National Center for Health Statistics (NCHS) and the Centers for Medicare and Medicaid Services External, Atlanta, GA, USA [16]. The HDRs also reported the modality of discharge of each patient, the modality of admission (emergency admission or programmed admission) and the type of the hospitalization (ordinary hospitalization or day-hospital stay). The length of stay was reported as the number of days calculated by the difference between the date of admission and the date of discharge. The cost of each hospitalization was also reported in the HDR: it was expressed in Euro (EUR) and referred to a fixed tariff relating to the specific DRG code. 

This study included data of hospitalizations that occurred among subjects aged from 1 day to 71 months and resident in the Abruzzo region. On the other hand, admissions occurring in one of the Abruzzo region hospitals by patients resident in another Italian region were excluded from the analysis. Only HDRs including the ICD-9-CM diagnosis code of 560.0 among any of the discharge diagnoses were included in the analysis. Repeated admissions were not excluded from the analysis.

### 2.2. Statistical Analysis

Descriptive continuous variables were summarized as mean and standard deviation (SD) or median and interquartile range (IQR) according to their distribution. Categorical variables were summarized as frequencies and percentages (%). A Kolmogorov–Smirnov test was performed to evaluate the normal distribution assumption of each continuous variable. Annual and total hospitalization rates relating to RV disease were estimated and expressed as per 10,000 inhabitants. Hospitalization rates were standardized for age and gender according to the Abruzzo population in the year 2015, the first year of the study. The joinpoint model, performed by Joinpoint version 4.6.0.0 (2018), was used to assess the time trends of the standardized rates and to calculate the average annual percentage change (AAPC). The AAPC represents a summary indicator of the trend over the time frame that is computed as a weighted mean of the annual percentage change (APC). The final model was developed as linear segments connected at joinpoints that demonstrated the best-fitting model of the observed data. Length of stay and cost variables were analyzed and compared with a Kruskal–Wallis test, with relative pairwise comparisons. For all analyses, a *p*-value less than 5% was considered statistically significant. Data about the demographic structure (gender and age) of the Abruzzo population in each year of the study were extracted from the Italian National Institute of Statistics (ISTAT) website. The statistical analysis was performed with STATA v14.2 software (StataCorp LLC, College Station, TX, USA).

### 2.3. Ethic Statement

The study was conducted in conformity with the regulations on data management of the Regional Health Authority of Abruzzo and with the Italian law on privacy (Art. 20-21 DL 196/2003), published in the Official Journal, n. 190, on 14 August 2004. The data were encrypted prior to the analysis at the regional statistical office when each patient was assigned a unique identifier. The identifiers eliminated the possibility of tracing the patients’ identities. According to Italian legislation, the use of administrative data does not require any written informed consent from patients.

## 3. Results

During the study period (2015–2021), a total of 664 patients aged less than 6 years were admitted to different hospitals for RV infection in the Abruzzo region. The distribution of cases was almost equal between genders: 355 (53.5%) cases were of the male gender and 309 (46.5%) were of the female gender. The most frequent age categories of patients admitted were below 1 year (164, 24.7%) and between 1 and 2 years (164, 24.7%), as shown in Table 1. Patients aged between 24 and 35 months numbered 117 and represented 17.6% of the total. Among older age groups, 92 patients (13.9%) were in the 36–47-month category, 66 were in the 48–59-month category (9.9%) and finally 61 patients (9.2%) were in the older group (60–71 months). The median LOS during the entire study period was 4 days (IQR 3–5). The median cost was EUR 1161.88 (1161.88–1620.99), based on the DRG code reported in the HDRs. One of the most crucial points of the study was that no patients underwent repeated hospitalization due to rotavirus. 

The age and gender standardized incidence rate showed an increase during the first five years of the study period, growing from 1.84 (95%CI 1.46–2.23) in 2015 to 3.63 (2.14–4.76) in 2019 with an annual percentage change (APC) of +13.9% (95%CI 12.6–15.2, *p*-value < 0.001), as shown in Table 2, with a linear increase during these years.

**Figure 1:**
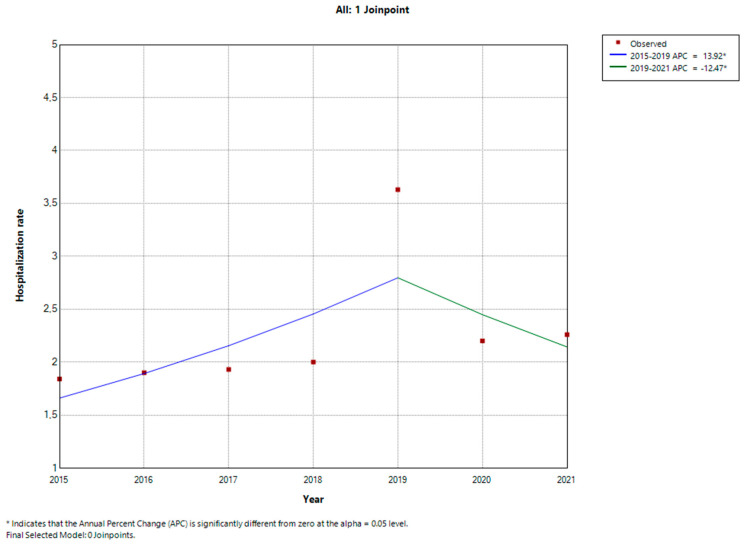
Trend in hospitalization rate shown by joinpoint regression model reporting Annual Percentage Change (APC).

The trend decreased during the years 2020 and 2021, which had an incidence of 2.20 (95%CI 1.52–2.88) and 2.26 (95%CI 1.72–2.79), respectively. The APC relating to the years 2020–2021 was −12.5% (95%CI −9.3–−15.5, *p*-value = 0.004). The joinpoint regression analysis with the APC is shown in Figure 1.

**Figure 2:**
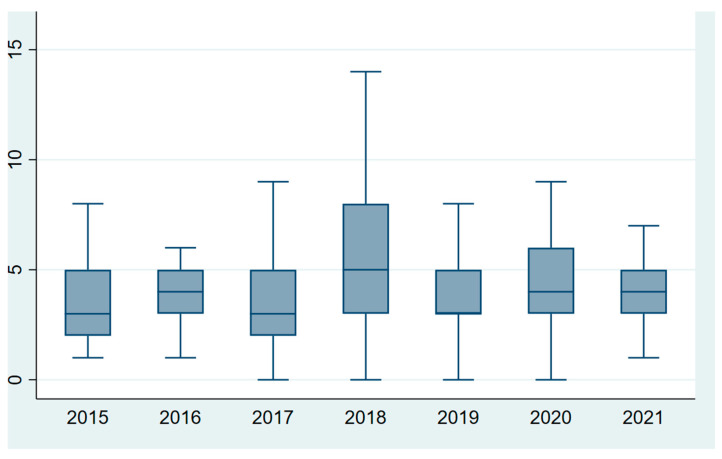
Length of stay distribution in days by year of age. Legend: the central mark indicates the median value and the bottom and top edges of the box indicate the 25th and 75th percentiles, respectively.

The length of stay was significantly different across the years, ranging from 3 days (2–5) in the year 2015 to 5 days (3–8) in the year 2017 (Kruskall–Wallis test *p*-value = 0.002), as shown in Figure 2. The year 2018 also showed a large variability in LOS, ranging from 1 to 14 days of admission.

**Figure 3:**
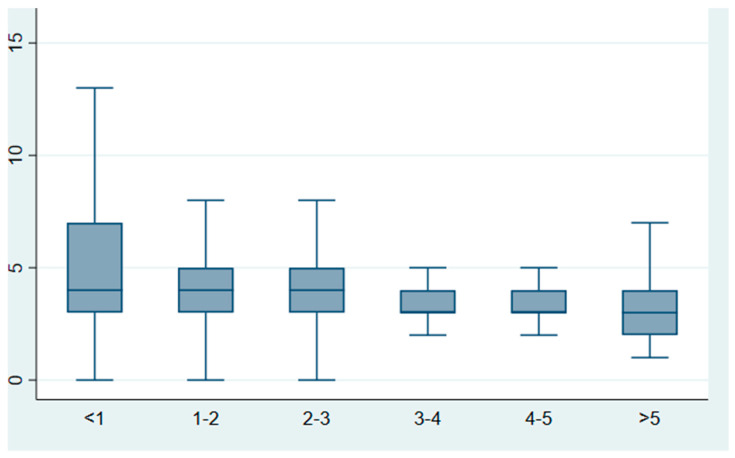
Length of stay in days by age group. Legend: the central mark indicates the median value and the bottom and top edges of the box indicate the 25th and 75th percentiles, respectively.

Length of stay was also significantly different across age group categories (Kruskall–Wallis test *p*-value < 0.001), as shown in Figure 3. In particular, patients aged less than 1 year had a median length of stay of 4 days (IQR 3–7) compared to patients aged between 3 and 5 years who had a median length of stay of 3 days (IQR 2–4). Pairwise comparisons showed length of stay of the younger age group was significantly different compared to all the older age groups evaluated in the study (pairwise comparison *p* < 0.001 for each comparison between <1 age group and all other groups). 

Regarding costs, the total amount of admission expenditure derived from the DRG codes during the study period was EUR 1.02 million. The median admission costs evaluated across the years was stable during the study period, as shown in Table 3. No differences between years were found in the statistical analysis.

## 4. Discussion

Rotavirus is the main cause of moderate to severe acute viral gastroenteritis in neonatal and pediatric patients. This was confirmed by several studies reporting that the great part of diarrhea and dehydration admissions in children were caused by rotavirus infection, particularly in low- and middle-income countries [12].

The present study was conducted in Abruzzo, an Italian region with suboptimal vaccination coverage against rotavirus [13,17]. During the study period, 664 patients were admitted to different hospitals for RV. The results showed an increasing trend until the year 2018 and a decreasing one during the last two years of the study period. The increasing trend was an opposite result compared to similar studies performed in Italy: both Dettori et al. [18] and Costantino et al. [12] reported a reducing trend during the same periods. This can be explained by the different introduction of RV vaccination in the Abruzzo region compared to other Italian regions. In particular, in the Abruzzo region, the vaccination was offered only from the year 2017. In February 2017, the Italian National Vaccination Plan (Piano Nazionale Prevenzione Vaccinale—PNPV 2017–2019) aimed to overcome regional differences by recommending universal rotavirus vaccination for all children over 6 weeks of age, free of charge. The objective of this plan was to increase vaccination coverage to ≥60% (2017), ≥75% (2018) and finally ≥95% (2019) [19]. Several studies reported that a significant reduction in rotavirus admissions was observed as vaccination coverage between 60% and 85% [20,21] was achieved, so improving vaccination coverage is a key factor to reduce the burden of rotavirus diseases. The global impact of rotavirus vaccination is evident, with a 40% reduction in rotavirus incidence demonstrated following the introduction of vaccines, as shown by a study performed in several countries participating in the Global Rotavirus Surveillance Network [22]. The absence of readmission during the study period suggests the strong immunogenic effect of severe rotavirus diseases in preventing further RV admissions.

Low vaccination coverage, influenced also by the fear of adverse vaccination reactions such as intussusception, can lead to an increasing trend in rotavirus admissions [23]. Also, poor knowledge of the disease can lead to skepticism towards RV vaccination: a survey of primary care pediatricians carried out in Italy during the year 2013 showed that only half of the respondents recommended RV vaccination [24]. In fact, a recent survey showed that being informed by physician was a significant strong predictor of willingness to get the rotavirus vaccination [17]. So, improving the knowledge of healthcare workers is a key factor in increasing vaccination coverage and in limiting the impact of infectious diseases on healthcare services. The evaluation of factors associated with vaccine hesitancy demonstrates that the socio-demographic parameters of parents were not significantly linked to willingness to get the rotavirus vaccination for their child. So, it is crucial to empower parents on vaccination importance. The lack of association between socioeconomic status and the risk of infection shows the need to focus the workforce on vaccination coverage improvement.

Increasing research shows that rotavirus infection is not limited to the gastrointestinal tract. In fact, infection can be present without diarrhea and may trigger neurological symptoms (such as seizures and epilepsy) and autoimmune diseases (such as diabetes mellitus and celiac disease), among others. These findings need further investigation, and they can offer new clinical perspectives on rotavirus vaccination and new opportunities for public health preventive measures [25,26]. It is well known that infants younger than 2 years of age have an increased risk of seizures associated with fever, so younger infants could also need hospital care for these reasons [26].

The decreasing trend reported during the years 2020–2021 may have an important explanation: while the improved vaccination coverage could have influenced the trend, the COVID-19 pandemic strongly impacted hospital admissions. In particular, both infectious diseases other than COVID-19 [27] and non-infectious diseases underwent a significant reduction in hospital admissions due to the pandemic [28,29,30,31]. Undoubtedly, the lockdown and the use of face masks helped in reducing the circulation of infectious agents in addition to SARS-CoV-2. In addition to the lockdown, several cities in the Abruzzo region experienced a prolonged school closure, also seen among kindergartens, during the pandemic period. This situation further limited rotavirus circulation and led to a decrease in the risk of contagion. The return to normal life with the circulation of all infectious pathogens will probably lead to an increase in rotavirus hospitalizations. It should be considered that in Italy rotavirus vaccination was only recommended to newborns and was not mandatory. During the lockdown, Italy observed a significant decrease in vaccine coverage for non-mandatory vaccination, and this point could play a role in a resurgence of rotavirus hospitalization after the year 2021 [27].

Regarding age, this study confirms a more frequent hospitalization rate due to RV among infants aged less than 2 years [32]. It should be also considered that, particularly among infants aged less than 1 year, the month of birth plays a key role in the risk of RV infection and related hospitalization: RV infection has a seasonal trend, so patients born during months with historically high rotavirus disease incidence have an increased risk of infection and hospitalization. On the other hand, patients born outside of the rotavirus season had a lower likelihood of being admitted for RV [32].

Regarding length of stay, this study showed that younger patients had longer hospital stays compared to other age groups. These findings are in line with the previous literature [32] and can be explained by the higher need for healthcare assistance for neonatal patients compared to older infants. In fact, the literature has shown that severe diseases occur more frequently among infants aged less than 1 year, highlighting the need to improve vaccination strategy gaining better vaccination coverage [5]. This point can be explained by the greater risk of dehydration due to diarrhea and vomiting of infants aged less than 1 year compared to older infants [32]. 

Admissions costs were reported to remain stable across the years. Certainly, the costs of hospital assistance for a single patient were not changed during recent years and the stability of length of stay may also explain the unchanged trend in hospital expenditure. It should be considered that the tariff of a single DRG code relating to rotavirus-related hospitalizations was not changed across the study period and this may also explain the unchanged trend. 

Finally, the potential impact of vaccination on hospital admissions for other gastroenteritis infections should also be considered, as reported by Mattei et al. [32].

### Strengths and Limitations

With the lack of a specific surveillance system, the use of HDRs represents an important tool to evaluate the impact of RV infection on healthcare services. One of the main points of strength of the present study was the use of standardized and routinely collected data from the entire population of an Italian southern region. To our knowledge, this is one of first studies conducted in Europe that covers the most recent years of rotavirus-related hospitalizations and that also considers the pandemic and post-pandemic periods. The evaluation of an entire population, substantially stable across years, can be used as a representative sample of the estimation of primary prevention interventions, such as a vaccination campaign, or it can be used as a valuable tool to assess the impact of infectious diseases on the hospitalization rate. In addition, this is one the first studies performed in the Italian region of Abruzzo on rotavirus incidence.

However, this study has several limitations. Firstly, it analyzes the burden of diseases of a single Italian region with a particular health system that applies preventive measures towards infectious diseases differently from other Italian regions.

The second limit is that the HDRs were not used for epidemiological scope but for remunerative purposes. So, for this reason, some information such as comorbidities may be underreported or miscoded.

Thirdly, HDRs do not contain patients’ clinical data, such as drug therapies, blood parameters and the clinical severity of the disease. Also, the vaccination status of each included patient was not available, not allowing the evaluation of the effectiveness of vaccination against rotavirus infection and to estimate its impact on the rotavirus admission trend.

Regarding costs, the HDRs reported a fixed tariff relating to the DRG linked to each admission, so this does not represent the real cost of each hospitalization. In addition, costs of out-of-hospital assistance, such as ambulatory care, domiciliary drug therapies and physician visits, could not be taken into account. So, the cost variable represents only an estimation of the real cost that occurred in assisting patients with rotavirus, limited to patients needing hospital care. In this context, the economic impact of rotavirus infection cannot take into account working days lost as consequences of the diseases, so the cost analysis needs a different source of data to be implemented.

## 5. Conclusions

Despite the introduction of mass vaccination, rotavirus represents a heavy burden for the healthcare system. The decreasing trend observed during the pandemic years should be maintained during subsequent years, particularly with the improve ment of vaccination coverage. HDRs represent a valid and useful tool to evaluate the direct impact of rotavirus disease on the pediatric population, in particular among neonatal patients.

## Figures and Tables

**Table 1:** Characteristics of patients admitted for rotavirus during years 2015–2021.

	*n* (%)
Gender	
Male	355 (53.5)
Female	309 (46.5)
Age group (months)	
<12	164 (24.7)
12–23	164 (24.7)
24–35	117 (17.6)
36–47	92 (13.9)
48–59	66 (9.9)
60–71	61 (9.2)
	median (IQR)
LOS	4 (3–5)
Costs in EUR	1161.88 (1161.88–1620.99)

LOS = Length of stay; IQR = Interquartile range.

**Table 2:** Standardized incidence of RV hospitalization by year/10,000 inhabitants.

Calendar Year	Incidence (95%CI) *
2015	1.84 (1.46–2.23)
2016	1.90 (1.21–2.58)
2017	1.93 (1.41–2.47)
2018	2.00 (1.41–2.46)
2019	3.63 (2.14–4.76)
2020	2.20 (1.52–2.88)
2021	2.26 (1.72–2.79)

* The incidence rate was standardized by age and gender.

**Table 3:** Cost of admissions by calendar year according to the tariffs expressed by DRG.

Calendar Year	Cost in EUR Median (IQR)
2015	1161.88 (1161.88–1161.88)
2016	1161.88 (1161.88–1500.07)
2017	1161.88 (1161.88–1620.99)
2018	1161.88 (933.69–1620.99)
2019	1161.88 (1092.12–1501.86)
2020	1161.88 (1118.63–1620.99)
2021	1190.00 (1190.00–1660.00)

IQR = Interquartile range; DRG = Diagnosis related group.

## Data Availability

Data are not available due to the restriction policy of the Abruzzo region.
